# Developing a desktop application for drug-drug interaction checker ordered for chronic diseases in Ethiopian hospitals pharmacy

**DOI:** 10.1186/s40360-022-00576-4

**Published:** 2022-06-06

**Authors:** Thamineni Bheema Lingaiah, Yibeltal Andarge Belay, Kokeb Dese

**Affiliations:** 1 School of Biomedical Engineering, Jimma Institute of Technology, Jimma University, 378, Jimma, Ethiopia; 2 Faculty of Computing, Jimma Institute of Technology, Jimma University, 378, Jimma, Ethiopia; 3 School of Computing and Informatics, Mizan Teppi University, Teppi, Ethiopia

**Keywords:** Drug-drug interaction, Chronic diseases, Desktop application, Medscape

## Abstract

**Background:**

Drug-drug interactions are a major cause of morbidity worldwide and are a leading source of treatment inefficacy. They are classified based on their pharmacological action on the body as major, moderate, and minor. Currently, it is a tedious process to remember the drug-drug interactions by the pharmacist during dispensing of the prescribed drugs for the patients. Therefore, there is a need for technology that assists the pharmacist in checking the drug-drug interaction for prescribed drugs. Therefore, in this work, a desktop-based application that can automatically identify the drug-drug interactions for prescribed drugs that could operate offline for those found in low-resource setting hospitals has been developed. To do this, around 3000 drugs along with their major and moderate interaction points were collected from Ethiopian Pharmaceutical Supply Agency. The developed system included two main parts; the database part that comprises all the drugs collected along with their major and moderate interaction points, and a patient registration platform to register the patients' history. The system was developed by using C sharp programing language.

**Results:**

The developed system has both drug-drug interaction checking as well as patient registration platform. Registration of the patient’s history will be done by the pharmacist and during dispensing of the drugs to the patient, the developed system will check the interaction between the drugs prescribed. The system was tested to operate the above functions, and finally, it was able to display the major and moderate interaction points of all inserted drugs automatically and accurately. For those drugs which have no either major or moderate interaction, the system was displayed as ‘*unknown*’.

**Conclusions:**

The developed system assisted the pharmacist in knowing the drug-drug interaction, and enabled the patients for the resubscription of drugs with the same functional. The system would help to increase the efficiency of the pharmacist in low resource settings to do their tasks without any difficulty, and tiredness. In the future, it is recommended to include all drugs for all disease types rather than focusing only on chronic disease drugs.

**Supplementary information:**

The online version contains supplementary material available at 10.1186/s40360-022-00576-4.

## Background

A drug interaction is a situation in which a substance (usually another drug) affects the activity of a drug when both are administered together. Drug-drug interactions (DDI) occur when two or more drugs react with each other and are a threat to public health [1]. This type of drug-drug interaction causes for about 2.8% of elderly patient to be hospitalized, where the government annual cost for financial healthcare system is estimated to exceed 1 billion US $ worldwide. [2, 3]. Previous studies focused on patients in emergency departments and hospital wards [4, 5] have shown that chronic patient drugs are the most common culprit of DDI. Adverse drug events (ADE) have become one of the main public health problems of concern to patients and healthcare professionals, and one of the specific types of ADE is drug interaction (DDI) [6]. Drug-drug interaction (DDI) is estimated to account for 3–5% of preventable adverse drug events (ADE) [7]. Drug-drug interaction is an important factor leading to various adverse consequences (such as adverse pharmacological reactions) [8, 9]. This drug-drug interaction may cause the user to get exposed to unexpected side effect. For example, mixing medications used to help sleep (sedatives) and medications used for allergies (antihistamines) can slow down your reaction and make driving a car or operating machinery dangerous [10].

At present, the number of comorbidities, multiple medications, and hospitalizations in Ethiopia has increased [11]. Therefore, the possibility of drug-drug interactions in the hospital is high. The prevalence of patients with potential drug interactions in Ethiopian hospitals was found to be 72.2% (95% confidence interval: 59.1, 85.3%). According to the severity, the incidence of potential major, moderate, and minor drug interactions were 25.1%, 52.8%, and 16.9%, respectively, and the incidence of contraindications was 1.27%. Factors related to possible drug-drug interactions are related to patient characteristics, such as multiple medications, age, comorbidities, and length of hospital stay where a large number of patients are hospitalized [12]. Therefore, the possibility of DDI is very high. In addition, due to economic issues, it is not feasible to use sophisticated instruments to monitor patients with comorbidities; leading to DDI in patients. Due to this, potential DDI poses a serious risk to the patient's health [12]. Drug-drug interactions are classified based on their pharmacological action on the body as major, moderate and minor, and the interactions may be due to pharmaceutical, pharmacokinetic, and pharmacodynamics [12, 13].

1. Major: Highly clinically significant and avoid combinations; the risk of the interaction outweighs the benefit.
2. Moderate: Moderately clinically significant. Usually, avoid combinations and use them only under special circumstances.
3. Minor: Minimally clinically significant. Minimize risk and can consider an alternative drug.

Regardless of the severity of DDI, patients should be monitored for possible manifestations of interactions [12]. Drug-drug interactions (DDIs) are a major cause of morbidity worldwide and a leading source of treatment inefficacy in Ethiopia. For this reason, DDIs cause great concern to patient safety and pharmacovigilance. According to [12], the incidence of drug interactions that may occur in Ethiopian hospitals is high. Medical workers (such as doctors, pharmacists, and nurses) remain vigilant in detecting, diagnosing, and reporting DDI, especially among at-risk individuals (such as patients with heart disease), which is also essential for continuous drug safety monitoring [6]. Previous studies focused on patients in emergency departments and hospital wards [4] have shown that chronic patient drugs are the most common culprit of DDI.

Pharmacists must take responsibility for monitoring drug interaction and notifying the physician and patient about potential problems**.** The lack of a drug-drug interaction assisting system during drug dispensing for outpatients is one of a challenge that increases the associated risks in low-resource settings. Therefore, the main objective of the research was to develop and formulate a desktop software application that can detect drug-drug interactions that assist the pharmacist during drug dispensing. In this research project, all drugs prescribed for chronic patients were collected from Ethiopian Pharmaceutical Supply Agency (EPSA) and local drug manufacturing companies, and automatic identification of drug-drug interactions systems has been developed.

A recorded data from Jimma University Medical Center (JUMC) shows that, if the pharmacists have known the pharmacology of the drug well enough, it is not much difficult for them to remember the interaction points of the drugs. However, the junior pharmacists who were usually assigned to work in low-resource settings, face difficulty in remembering the interaction level of drugs. During this time, manually referring to electronic or printed sources about the drug-drug interaction level is become tedious and time-consuming. Our system is developed to solve such difficulties and help to assist the pharmacist. The developed system provides a fast and automatic response to the drug's interaction level. For the patient registration, it took an average of a minute, and to select the drug from the database and check the drug interaction level, an additional average of a minute was spent. Therefore, a total of an average of two minutes were depleted to make the patient registration and drug-drug interaction checking. Nevertheless, if the patients were registered once, no need for re-registration on the next visit. Instead, it is possible to put out the patient's history using the ID number or the first name of the patient. This would help to serve the patients efficiently within a minute. However, presently, a large number of drugs are introduced every year, and new interactions between medications are increasingly reported. Consequently, it is no longer practical for a pharmacist to rely on memory alone to avoid potential drug interactions. Therefore, by upgrading our system within a period, it is possible to assist pharmacists in their work.

### Implementation

Before developing the software-based desktop application, a national survey of approved drugs with their type of strengths, brand names with routes, and their drug interactions was conducted. This study contributed to controlling the non-approved and counterfeit medications in the Ethiopian market. Moreover, it is a single platform to counter-check approved drugs by Ethiopian Pharmaceutical Supply Agency (EPSA). EPSA is a legal entity established under the law of the Federal Democratic Republic of Ethiopia Government to overcome the problems and assure uninterrupted supply of pharmaceuticals to the public at an affordable price.

### National wide survey drug collection

There are vast numbers of medicines in the present market. Availing all brands of medicine was never cost-effective. Ensuring the availability of safe, effective, and good quality medicines should control and limit the number of drugs used. Ethiopian drug policy listed the national essential drug list along with its therapeutic usages, which are required for the prevention, diagnosis, treatment, mitigation, and rehabilitation of diseases that affects the majority of people in service of quality delivery. Ethiopian Pharmaceutical Supply Agency (EPSA) is of quality control agency established by the Minister of Health (MOH) to assess the quality of medicines, which are locally manufactured and imported. The EPSA recommends the ANDA (A New Drug Application) applicants for market approvals to submit their product label information. A descriptive-based national-wide study was carried out with the help of structured questionnaires for the drugs used for chronic diseases in Ethiopia. The pharmacists which were participated as data collectors have registered around 3,000 drugs given to chronic disease patients along with their drug-drug interaction points. Pharmacological Books, Product Information, Pub Med, EBSCO Information Service, Drugs.com, and National Institute of health electronic databases were searched to identify the drug-drug interaction information. Once the correct drug interaction level had been collected accurately, it was filled in the Microsoft Excel sheet (see sample drugs in Table 1). Then, each interaction point and level has been carefully coded in the database. Coding of each drug along with their interaction point had been done with great caution. Because any wrong information coding leads to false output.

**Table 1:** Sample drug names with their interaction points

S.no	Drug name	Major drug interaction	Moderate drug interaction
1	Acetazolamide	Duloxetine	Topiramate
2	Acetylsalicylic acid	-	Gentamicin
3	Dexamethasone	Ciprofloxacin	
4	Fluconazole	-	Ciprofloxacin
5	Furosemide	-	Carbidopa + levodopa
6	Mipomersen	Acetylcysteine	
7	Pregabalin	-	Cetirizine dihydrochloride
8	Salmeterol	-	Ciprofloxacin
9	Abiraterone	amiodarone, amisulpride, anagrelide	-
10	Acitretin	aminolevulinic acid, bexarotene	-
11	Adalimumab	Isoniazid	-
12	Adalimumab	Methotrexate	Ethambutol
13	Adefovir	-	Celecoxib
14	Amikacin	Aspirin	
15	Aminophylline	Phenytoin	
16	Amiodarone	Carvedilol	warfarin
17	Amoxicillin + Clavulanate	-	Erythromycin
18	Amphetamine	-	salbutamol
19	Aspirin	Ibuprofen	Betametasone
20	Tramadol	Lidocaine	Warfarin
21	Sertraline	Amitriptyline HCl	Indomethacin
22	Ondansetron	Acetaminophen / hydrocodone	Duloxetine
23	Phenytoin	Lopinavir + Ritonavir	Ketoconazol
24	Piperazine	Amifampridine	Iohexol
25	Prednisone	Acetylsalicylic acid	Methotrexate

The data which was collected has been categorized based on the pharmacological classification system. All the drugs locally manufactured and imported were used as a source of population. Regarding the sampling and sample size determination, there was no specific equation matching for this type of study and does not have any earlier studies available. Therefore, we tried to include all the medications approved by EPSA, and the data missed from the regulatory agency, have been looked for support from the medical importers and manufacturers for completing the data collection. A systematic random selection for medical importers was used for data collection as it is difficult to include all. Different ANDA application approvals were reviewed and the registered drugs were collected using the pre-structured questionnaires. All drugs with their brand names, strengths, and type of dosage forms along with their interaction drugs were recorded. Regrading the data processing, the recorded data were categorized according to therapeutic classification in MS Office Excel sheet, the description of the study was analyzed, and the frequency of drugs that were approved per year has been presented in frequencies.

All the dosage forms were either imported or locally produced and the same drug with different brand names and different strengths were our inclusion criteria. This research excluded the medical devices and ANDA submission for the same brand name and same strength. In addition to this, it will avoid the recurrent approvals on the same drug product from different manufacturers on an existing brand name and excludes the same product with different brand names from the same manufacturer. Sample drugs collected along with their interaction point and level were attached in Table 1.

### Developing the drug-drug interaction desktop application

**Fig. 1:**
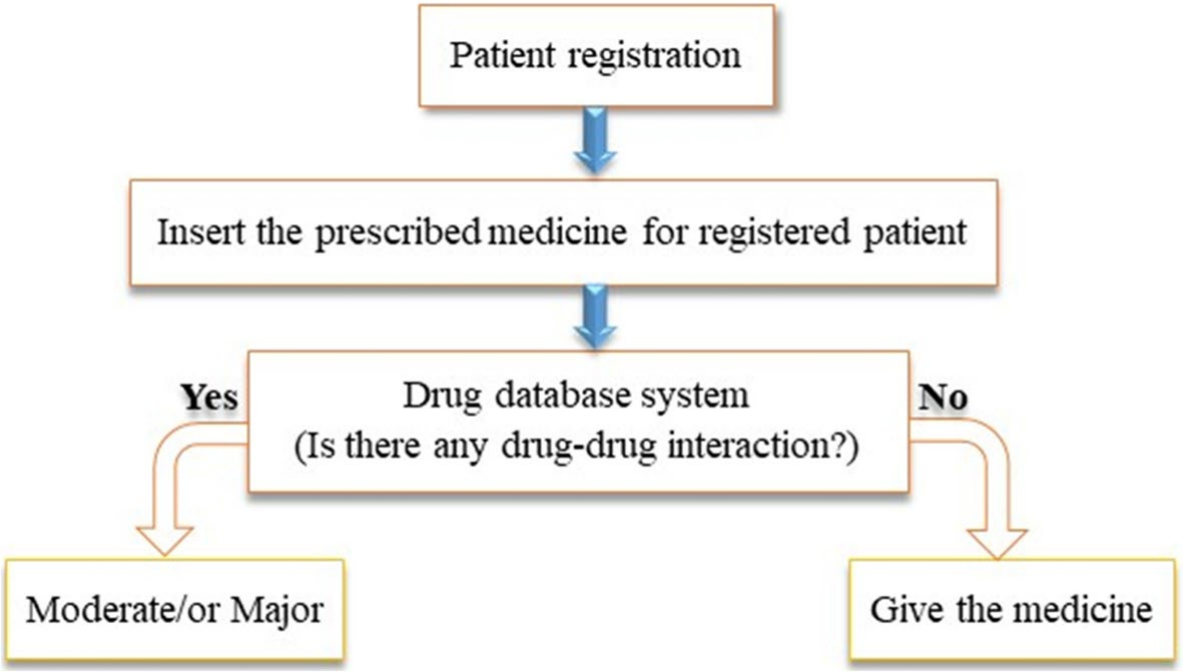
System workflow for patient registration and drug-drug interaction checker

The developed system includes two main platforms, (1) the database page, which includes all the drugs collected along with their interaction points and levels, and (2) a patient registration system, which helps to register the patient’s history while dispensing the drugs. The database system was created to store all the drug information and identify their interaction points and levels. Figure 1 shows the flow diagram of the developed system.

As depicted in Fig. 1, when patients go to a pharmacist to take a medicine, they must first register. Then the pharmacist verifies if the prescribed drugs have any drug-drug interaction point before dispensing them to the patient. If the system detects either a moderate or major drug-drug interaction, it would automatically pop up to display the interaction results. This helps the pharmacist to order a drug substitution/re-prescription for the treatment. Due to its simplicity and effectiveness, the entire system was developed by using C sharp programming language. For security reasons, the system has only one administrator/pharmacist login window. The administrator/pharmacist can then register the patient. The patient registration form includes the first name, middle name, last name, sex, age, patient card no/ID, mobile number, and email address of the patient. Pharmacists must take responsibility for monitoring drug interaction and notifying the physician and patient about potential problems. Therefore, whenever, our system is installed, before starting an operation enough training would be provided to the pharmacist working in the hospital or health center.

## Results

From the national wide survey, 3000 drugs along with their drug-drug interaction points were identified and collected, and both the database and the patient registration system were developed. The following section depicts step-by-step results obtained while developing the desktop software application. The study place was at Jimma University Medical Center (JUMC).

### Home page

**Fig. 2:**
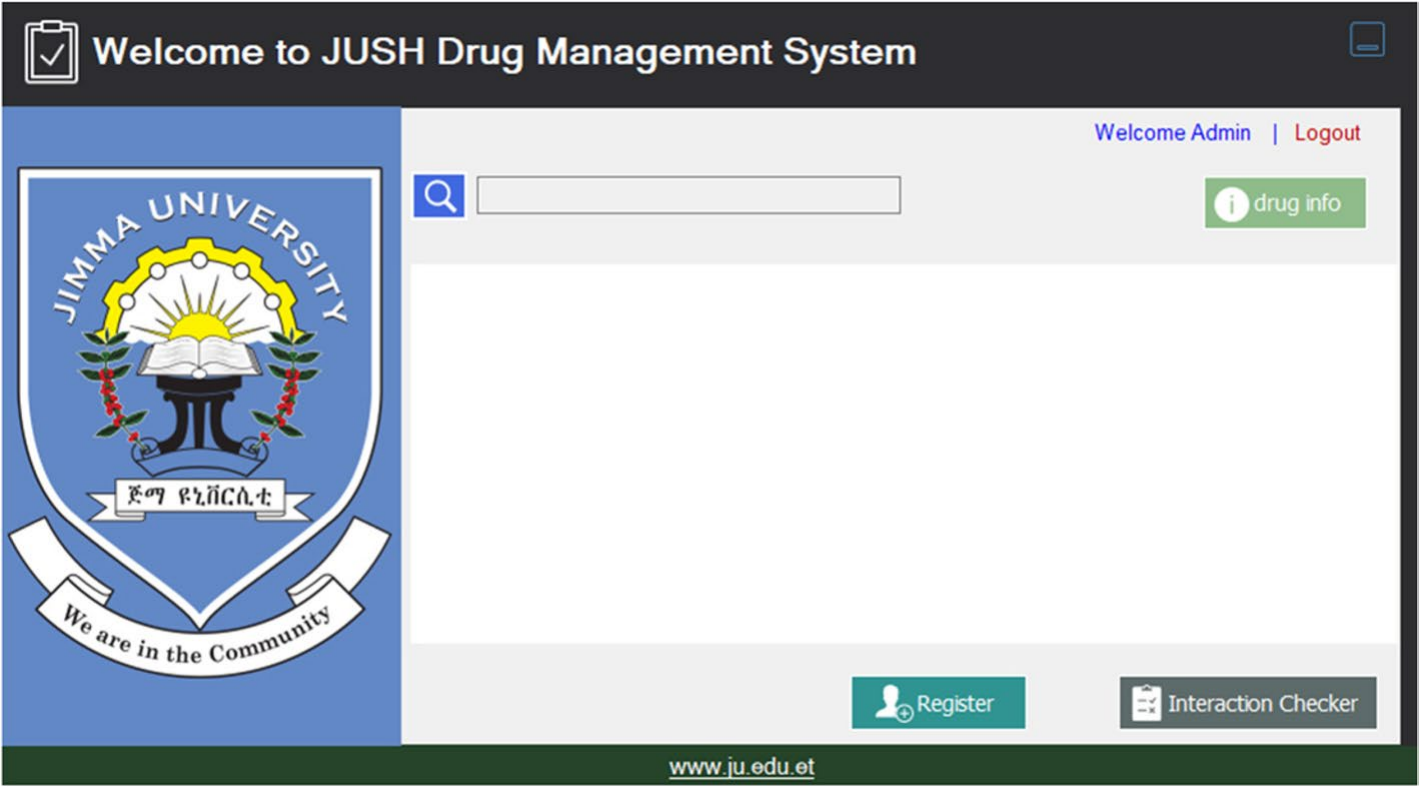
Home page of the system

This is the first page seen when the user/pharmacist opened the software application (see Fig. 2). It is the main page of the system. It provides information about the authenciated organization which has developed this application.

### Login page for pharmacist

**Fig. 3:**
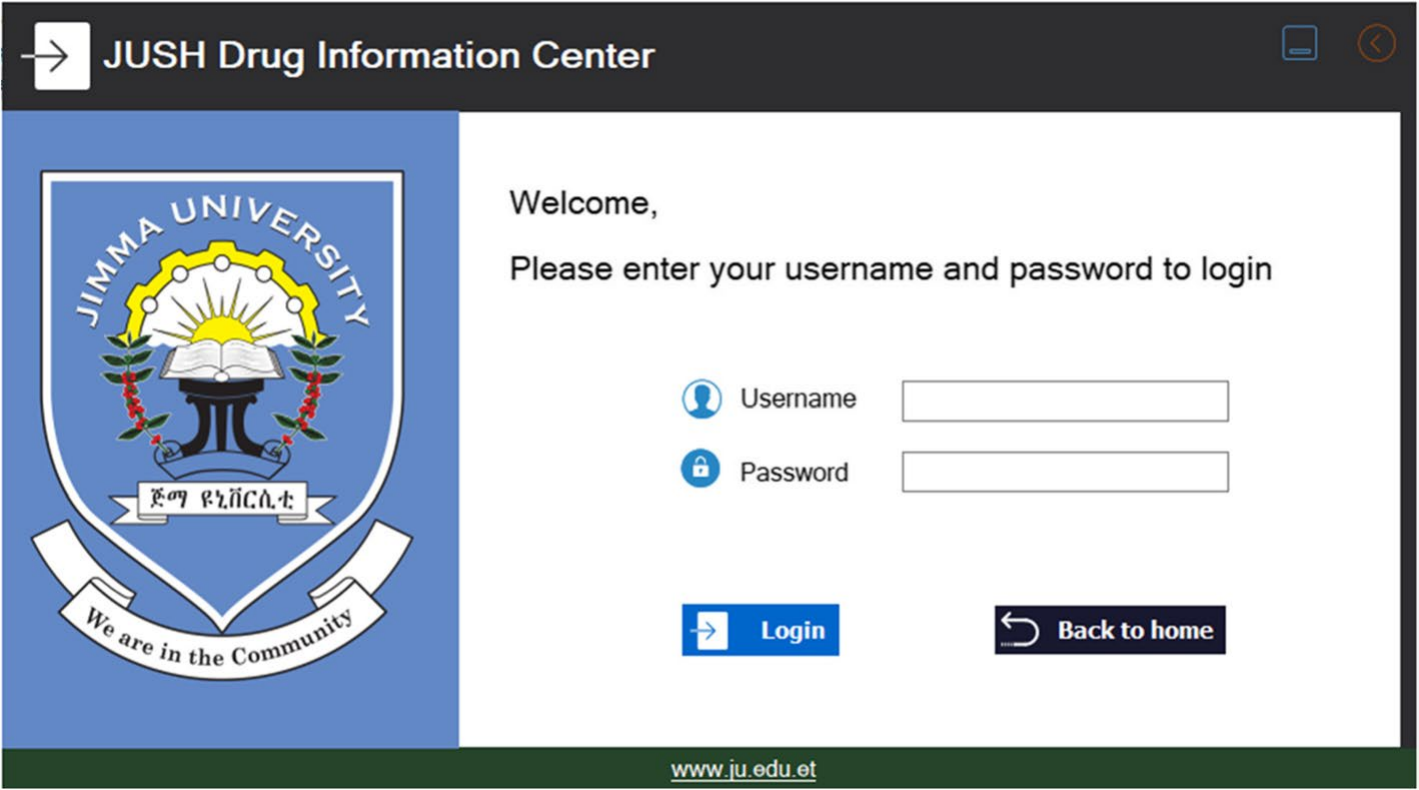
Login page for the authorized person

The second screen is the admin/pharmacist page(see Fig. 3). Admin person could be the pharmacist who uses the software. A login is a set of credentials used to verify the identity of a user. In most cases, these consist of a username and password. It is a security measure designed to prevent unauthorized access to confidential data. When the login fails (that is, the username and password combination do not match the user account), the user has denied access. In this system, only when the pharmacist would insert the correct username and password, could able to proceed to the next level/page. Any pharmacist could use the system with a correct password and user name. However, since our software system includes patients’ information, and due to ethical issues, it is advisable to keep the password and user name to be confidential with only accredited pharmacists who are working on the system.

### Patient registration form

**Fig. 4:**
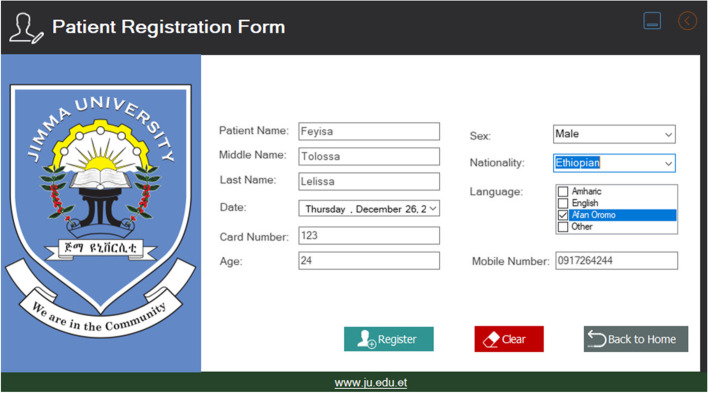
Patient registration form

The third one is the patient registration page. This page is shown when the pharmacist inserted his /her user name and password correctly. On this page, the pharmacist would register all necessary information about the patient’s history. As shown in Fig. 4 this included the patient's first name, middle name, last name, sex, age, patient card no/ID, mobile number, and email address.

### Drug-drug interaction checker page

**Fig. 5:**
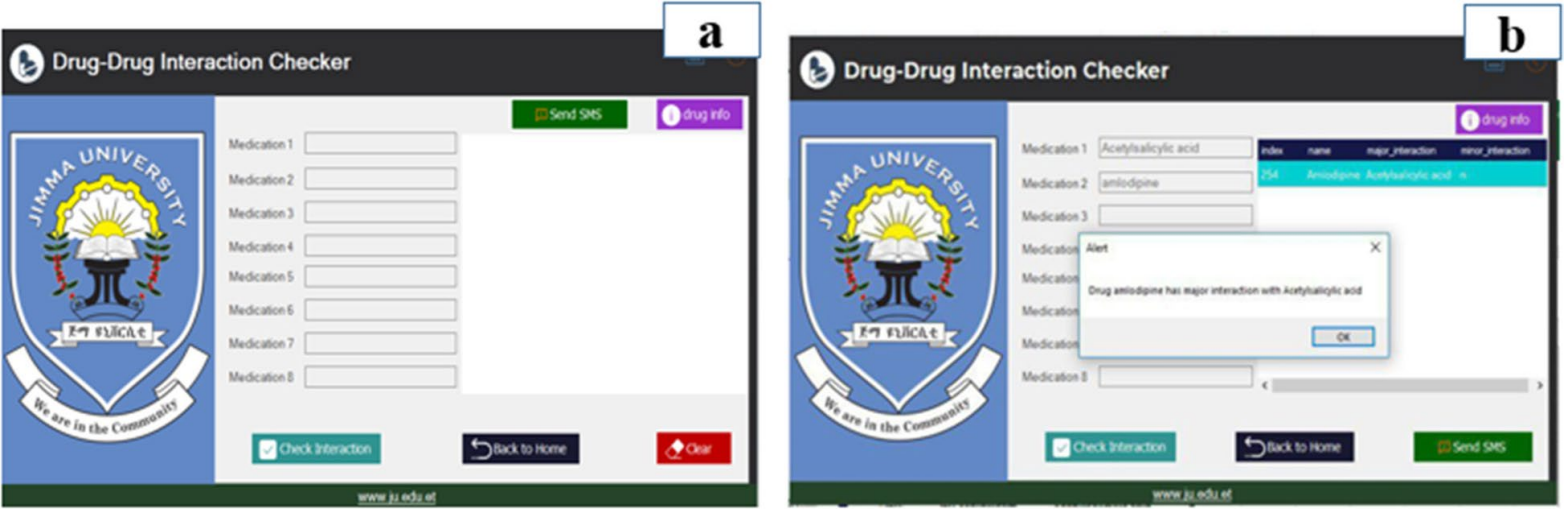
Drug-drug interaction checker page

Once the patient registration is over, the next step was be inserting all the drugs prescribed by the physician. To do this the pharmacist needs to open the interaction checker button from the previous page. When the button was clicked, Jimma University Specialized Hospital (JUSH) Drug-Drug Interaction Checker page was displayed as shown in Fig. 5a. Therefore, for the system to check whether the prescribed drug has major or moderate drug-drug interaction, the pharmacist had to enter the entire drug's name into the system. Once the drug names were correctly inserted, by clicking the ‘Check Interaction’ button it would be possible to see the major and moderate drug interaction points of the drug.

Figure 5b shows a sample example that the pharmacist inserted into the system and the system displayed an interaction point detected. Here the pharmacist inserted Acetylsalicylic acid and Amlodipine into the system. Then, when he pressed the drug interaction checker button it was displayed that both drugs have major interaction points. In addition to the above, the system also integrates a drug information system, which is linked with the drugs.com website.

Like any other software, our desktop application software system also needs routine preventive, corrective, adaptive, and perfective types of maintenance. Software maintenance is a core process in changing, modifying, and updating software to keep up with customer needs. Therefore, we would provide preventive maintenance quarterly to make changes and adaptations to our software so that it can work for a longer time. Adaptive maintenance would be done whenever there is a change in the operating system of the desktop computer. Correct software maintenance addresses the errors and faults within software applications that could influence various parts of our software, including the design, logic, and code. This would be done whenever their error happened on the system and enquired by the user. Perfective software maintenance focuses on the evolution of requirements and features that exist in our system. As users interact with our applications, they may notice things that we did not or suggest new features that they would like as part of the software, which could become future projects or enhancements. Therefore, this would be done once a year by the team.

Some of the error alerting features have been included in our developed software. For instance, if any drug which is not included in the database if inserted by the pharmacist, it would be displayed as an ‘*unknown drug’*. In addition to this, if the system is facing a failure at the patient registration or drug-drug interaction stage, it pops up as a ‘*patient registration error’* or ‘*fails to check the interaction*’ respectively. This would help the pharmacist to communicate with the developing team /developer or any software expert for the maintenance of the system.

## Discussion

Treatment options are becoming more complex and the risk of drug interactions increases. Drug interactions can cause significant harm to patients. This is due to drug toxicity or loss of efficacy. For example, voriconazole 1 and clarithromycin increase simvastatin concentration and the risk of rhabdomyolysis, while rifampicin reduces the anticoagulant effect of warfarin. Sometimes an interacting drug is deliberately co-prescribed. For example, diltiazem can be used to increase cyclosporine levels. Supplementary drugs can also cause drug interactions [14]. Clinicians should use the available resources on drug interactions, but remember that although the recommendations for each resource may be similar, there will be differences. It is important to assess the clinical significance of potential drug interactions and their relevance to each patient. To determine the interaction, you must first accurately list the patient's prescription drugs, supplements, and over-the-counter drugs. The other forms of administration, such as topical administration and administration by inhalation should also be considered [12, 15].

Prevention of DDI, especially for chronic patients, should be included in the pharmacological action plan, not only to assess DDI, but also to assess disease-drug interactions, identify and reconsider high-risk treatments, and adjust organ resection [7]. The Australian Medicines Manual (AMH) provides practical information on drug interactions that are considered clinically important. When appropriate, it provides specific information on drug metabolism but does not include main references. Interaction checkers MIMS and Australian Drug Information (AusDI), Stockley's Drug Interactions, and Lexicomp have assigned their own ‘severity/risk level’ or ‘importance’ to the interaction. They provide possible interaction mechanisms, recommended actions, and include clinical evidence and support, or in some cases controversial references [12].

Based on the information used and the criteria of editing, these resources can provide other advice on interactions, especially when specifying clinical significance.

Occasionally, interaction information is extrapolated with other drugs in the same series or similar drugs in metabolism. Interactions may not be included in the resource until they are reported to the TGA or published in a case report. Although the available resources generally provide information about the interaction between the two drugs, the patient has a lot of potential interactions and can take multiple drugs. Currently, there are no resources that can provide information about the overall risk of interaction with various drug combinations. Pharmaceutical information and pharmacists can provide advice in such cases. Moreover, the well-known online drug-drug interaction checker Medscape [1] provides a good drug-drug interaction point for all drugs. However, all the aforementioned software is only accessed online using the internet and does not include drugs manufactured locally. Due to this, the system is currently not easily accessible and affordable in low resource settings of Ethiopia. However, our system is focused on all the drugs available in local markets and distributed in all hospitals. There is no requirement for the internet to operate the system. Since almost all drugs given to chronic patients along with their drug-drug interaction points used in Ethiopia were included in the database of the developed system it would have a significant impact in reducing the risk associated with the drug-drug interaction effect and assisting the pharmacist in low resource settings. The developed system helps the pharmacist to easily identify the drug-drug interactions based on the prescribed drugs within a minute and protects the patients from the drug-drug interaction effect. Moreover, it is an user friendly, and low-cost system. However, one of the big challenges in low resource settings was the shortage and problem of electric power in health centers. Our system operates on a desktop computer which functions using electric power. Nevertheless, we had suggested using solar power for such low-resource settings where electric power is scarce. However, in the lack of either electric or solar power that helps to operate the desktop computer on which our system is installed, it is not possible to operate our software application. In addition to this, during the time by which the system needs serious corrective maintenance, it would stop functioning until maintenance is done to the system. Moreover, to minimize the cost, our team has focused on developing a software application that is only used for chronic disease drugs; therefore, it cannot be used for other types of disease drugs.

## Conclusions

Currently, it is a tedious process to recall all the drug-drug interactions by the clinical pharmacist. Therefore, our system can screen all the chronic diseases and drug interaction points easily from the database. The developed system automatically checks for the presence of major and moderate drug-drug interactions. This helps the pharmacist to easily control and monitor the drug-drug interaction. Therefore, the system would assist the pharmacist in controlling the risk that happened due to drug-drug interaction with the patient. This would intern helps the patients to protect their health from drug-drug interaction side effects. Collecting all disease drugs to develop a drug interaction for all diseases was much expensive. However, in the future, it is recommended to upgrade our software system to include all diseases and drug-drug interactions and to integrate a system that can send short message services about the drug instruction for the patients to improve the medication adherence that usually affects most patients.

## Availability and requirements

**Project name:** Desktop Application for Drug-Drug Interaction Checker

**Project home page: **It is a desktop application and can be found at http://doi.org/10.17605/OSF.IO/G5R7T

**Operating system(s):** Windows based operating system

Programming language: C#

**License:** GNU Lesser General Public License (LGPL) 3.0

**Other requirements:** window 7 and above operating system

Any restrictions to use by non-academics: license needed

## Supplementary information

**Additional file 1.**

## Data Availability

The developed system can be found in Open Science Framework (OSF) database at http://doi.org/10.17605/OSF.IO/G5R7T. However, the datasets for all the drugs collected along with their interaction point used during the current study are available from the corresponding author upon reasonable request.

## Abbreviations

ADE: Adverse drug events
AMH: Australian Medicines Manual
ANDA: A New Drug Application
AMH: Australian Medicines Manual
DDI: Drug-drug interactions
EPSA: Ethiopian Pharmaceutical Supply Agency
ID: Identification Card
JUSH: Jimma University Specialized Hospital
MOH: Minister of Health
MIMS: Monthly Index of Medical Specialties
NHI-IC: National Health Insurance Identification Card
TGA: Therapeutic Goods Administration
