# Supplementary material for: Developing a desktop application for drug-drug interaction checker ordered for chronic diseases in Ethiopian hospitals pharmacy
Source: BMC Pharmacol Toxicol. 2022 Jun 6;23:36. doi: 10.1186/s40360-022-00576-4 (PMC9169384; doi:10.1186/s40360-022-00576-4)
Supplement: Supplementary file 1 — Additional file 1. [file 40360_2022_576_MOESM1_ESM.docx]

**Supplementary Information**

The software application can be found at: <http://doi.org/10.17605/OSF.IO/G5R7T>

Instructions:

1. Download the three files
2. Read the ‘*How to operate the system’* file before running the application.
3. In addition to this, you can watch the short video attached to know more about on how to use the system.
